# Evaluating Long-Term Neurological and Functional Recovery in Acute Traumatic Spinal Cord Injury: A Prospective Cohort Study

**DOI:** 10.7759/cureus.95289

**Published:** 2025-10-24

**Authors:** Muhammad Tayyab, Muhammad Sajjad, Mahmood Ahmad, Naeem ul Haq, Zawar Ahmad, Asif Afridi, Muhammad Tanveer

**Affiliations:** 1 Department of Trauma and Orthopaedics, Bradford Teaching Hospitals NHS Foundation Trust, Bradford, GBR; 2 Department of Neurosurgery, King Fahd Military Medical Complex-Dhahran, Dhahran, SAU; 3 Department of Trauma and Orthopaedics, Milton Keynes University Hospital, Milton Keynes, GBR; 4 Department of Neurosurgery, Bacha Khan Medical College, Mardan, PAK; 5 Department of Trauma and Orthopaedics, Kettering General Hospital, Kettering, GBR; 6 Department of Trauma and Orthopaedics, Hayatabad Medical Complex Peshawar, Peshawar, PAK; 7 Department of Trauma and Orthopaedics, Royal Stoke University Hospital, Stoke-on-Trent, GBR

**Keywords:** asia scale, functional outcome, neurological recovery, rehabilitation, scim, spinal cord injury

## Abstract

Background

Traumatic spinal cord injuries often result in significant disability and impact patients’ functional independence and quality of life.

Objective

This study aims to evaluate neurological recovery, independence in daily activities, and overall physical performance in trauma patients with spinal injury over a 24-month follow-up, with a focus on outcomes in low-resource settings.

Methodology

A prospective descriptive observational study was conducted from January 19, 2022, to December 19, 2024. A total of 216 adult patients with acute traumatic spinal injuries were enrolled. Neurological status was assessed using the American Spinal Injury Association (ASIA) Impairment Scale, including motor and sensory subscores, and functional independence was measured with the Spinal Cord Independence Measure (SCIM) at baseline, discharge, and at six, 12, 18, and 24 months post-injury. Data on acute management, complications, specific rehabilitation therapies (physical therapy, occupational therapy, ambulation training), ambulation, and occupational reintegration were collected. Statistical analysis included repeated measures analysis of variance (ANOVA) and Chi-square tests, with significance set at p < 0.05.

Results

At baseline, 42.59% (n = 92) of patients had severe neurological impairment (ASIA A/B), which decreased to 21.30% (n = 46) at 24 months. The mean SCIM scores improved significantly from 38.60 ± 12.40 at admission to 74.90 ± 15.80 at two years (p < 0.001). Surgical intervention was performed in 68.52% (n = 148) of patients. Independent ambulation was achieved by 38.89% (n = 84), while 41.20% (n = 89) were unemployed due to disability. Common complications included pressure ulcers (11.57%, n = 25) and urinary tract infections (13.89%, n = 30) during hospitalization.

Conclusion

Significant neurological and functional improvements occur over 24 months, though complications and occupational challenges persist, highlighting the need for cautious interpretation due to heterogeneity in injury levels and rehabilitation protocols, and the importance of optimized rehabilitation in low-resource settings.

## Introduction

One of the most severe effects of trauma is spinal cord injuries (SCIs), which can lead to permanent disability, a worse quality of life, and a significant socioeconomic burden [1]. Road traffic accidents, falls from heights, and violence are the main causes of traumatic spinal injuries, which vary greatly in occurrence worldwide, ranging from 10 to over 80 cases per million people yearly [2]. Severe spinal injuries and related problems are far more likely to occur in low- and middle-income nations, where occupational laws, trauma care systems, and traffic safety measures are often lacking [3].

Both main mechanical insult and secondary injury processes, including ischemia, inflammation, and apoptosis, are part of the pathophysiology of SCI and may worsen brain damage [4]. Autonomic instability, bladder and bowel dysfunction, and partial or whole loss of motor and sensory abilities are all possible outcomes of these injuries [5]. The degree, intensity, promptness, and availability of specialist rehabilitation treatments all have a significant impact on recovery prospects [6].

Survival rates and functional recovery in individuals with SCI have increased in recent decades because of developments in emergency trauma treatment, surgical stabilization methods, and extensive rehabilitation programs [7]. Nevertheless, many people still face major barriers to their mobility, independence, and ability to participate in everyday activities in spite of these advancements [8]. Thus, functional outcome assessment, which includes motor recovery, self-care skills, and reintegration into social and vocational roles, has emerged as a crucial element in assessing the overall efficacy of trauma and rehabilitation treatments [9].

A number of instruments have been used extensively to measure functional outcomes, such as the Spinal Cord Independence Measure (SCIM), Functional Independence Measure (FIM), and American Spinal Injury Association (ASIA) Impairment Scale [10,11]. These tools help researchers find variables that affect long-term prognosis in addition to helping therapists track healing [12].

The difference between acute treatment and long-term functional recovery is still significant in areas with few rehabilitation resources. Customizing rehabilitation techniques, allocating resources optimally, and enhancing patient-centered care all depend on an understanding of functional outcomes in such settings. This study evaluated the functional outcomes in trauma patients with spinal injury, assessing neurological recovery, independence in daily activities, and overall physical performance after treatment and rehabilitation.

## Materials and methods

Study design and setting

This descriptive observational study was conducted over a three-year period from 19 January 2022 to 19 December 2024 at the Department of Neurosurgery, Mardan Medical Complex, Mardan.

Inclusion and exclusion criteria

Adult patients who were 18 years of age or older and who had acute traumatic spinal injury as determined by radiological imaging and clinical evaluation were included in the research. Enrollment was limited to patients who gave their informed consent and were willing to participate. Exclusion criteria included patients with significant concurrent injuries or comorbidities that hindered follow-up, as well as those with non-traumatic spinal cord diseases, such as tumors, infections, or degenerative illnesses. Additionally, those who were lost to follow-up prior to hospital release were not included in the study.

Sample size

Convenience sampling was used to select 240 individuals from among the eligible trauma patients who presented with acute spinal injuries. The single-center design and the goal of including all consecutive patients who satisfied the inclusion criteria over the three-year study period served as justifications for the convenience sampling strategy. Because the study's goals were exploratory and to replicate actual clinical procedures in a tertiary care context, no formal a priori power or sample size calculation was carried out. A total of 216 patients were available for final analysis due to loss to follow-up. Compared to previous real-world observational studies evaluating functional outcomes after spinal injury, the final sample size is similar [13,14]. The debate has recognized this restriction.

Data collection

A standardized proforma developed by the neurosurgical team was used to collect data prospectively, with baseline assessments performed within 72 hours of injury. Information on demographics, mechanism of injury, level and completeness of injury (assessed using the SCIM, version III (SCIM III) [15,16], for which formal permission was obtained from Prof. Amiram Catz of the Loewenstein Rehabilitation Medical Center and is provided in the Appendices, and the ASIA Impairment Scale, including motor and sensory subscores, comorbidities, and acute management details was recorded at baseline and during hospitalization. Rehabilitation details were also collected, including type, frequency, and duration of physical therapy, occupational therapy, and use of assistive devices. At discharge, neurological status (ASIA), functional status (SCIM III), hospital stay duration, and in-hospital complications were documented. Longitudinal follow-up at six, 12, 18, and 24 months included reassessment of neurological recovery, functional improvement, complications, rehabilitation progress, use of assistive devices, and reintegration into social and occupational activities, performed primarily through in-person visits or, when not feasible, structured telephone interviews.

Statistical analysis

IBM SPSS Statistics for Windows, Version 26 (Released 2018; IBM Corp., Armonk, New York, United States) was used to input and analyze all of the data. While categorical data were shown as frequencies and percentages, continuous variables were represented as mean ± standard deviation (SD) or median with interquartile range (IQR). Repeated measures analysis of variance (ANOVA) was used to evaluate changes in functional scores at many follow-up time intervals (baseline, six months, 12 months, 18 months, and 24 months). The Chi-square test was used to investigate relationships between category data. Where appropriate, post-hoc pairwise comparisons were performed using the Bonferroni correction. P-values below 0.05 were regarded as statistically significant.

Ethical approval

The study was approved by the Institutional Review Board (IRB) of Mardan Medical Complex Mardan, under approval no. 791, dated: 15/12/2021. Written informed consent was obtained from all participants prior to enrollment in the study.

## Results

The majority of the 216 individuals that were examined were between the ages of 18 and 29 (29.63%, n = 64) (Table 1). The cohort was dominated by men, making up 75.00% (n = 162). The primary cause of injury was automobile accidents (51.85%, n = 112), which were followed by falls from a height (36.57%, n = 79). The most prevalent injury levels were lumbar (25.93%, n = 56), cervical (31.48%, n = 68), and thoracic (42.59%, n = 92). While 17.59% (n = 38) and 10.19% (n = 22) of patients had diabetes mellitus and hypertension, respectively, two-thirds (67.13%, n = 145) did not have any comorbidities.

**Table 1:** Baseline demographic and clinical characteristics

Variable	Category	Number of patients (n;%)
Age group (years)	18-29	64 (29.63)
	30-39	51 (23.61)
	40-49	47 (21.76)
	≥50	54 (25.00)
Gender	Male	162 (75.00)
	Female	54 (25.00)
Mechanism of injury	Road traffic accident	112 (51.85)
	Fall from height	79 (36.57)
	Violence/assault	15 (6.94)
	Sports/Other	10 (4.63)
Level of injury	Cervical	68 (31.48)
	Thoracic	92 (42.59)
	Lumbar	56 (25.93)
Comorbidities	None	145 (67.13)
	Hypertension	38 (17.59)
	Diabetes mellitus	22 (10.19)
	Others	11 (5.09)

The 24-month follow-up showed a substantial improvement in functional outcomes. After baseline, the mean SCIM score was 38.60 ± 12.40; after 24 months, it was 74.90 ± 15.80 (p < 0.001) shown in Table 2. While the percentage of patients with improved neurological status (ASIA C/D/E) rose from 57.41% (n = 124) to 78.70% (n = 170), the percentage of patients with severe neurological impairment (ASIA A/B) dropped from 42.59% (n = 92) at admission to 21.30% (n = 46) after 24 months.

**Table 2:** Neurological and functional status over time SCIM: Spinal Cord Independence Measure; ASIA: American Spinal Injury Association

Stage	Mean SCIM score ± SD	ASIA A/B (%)	ASIA C/D/E (%)
Baseline (admission)	38.6 ± 12.4	92 (42.59)	124 (57.41)
Discharge*	45.0 ± 13.0	85 (39.35)	131 (60.65)
6 months	52.1 ± 13.7	78 (36.11)	138 (63.89)
12 months	61.8 ± 14.2	63 (29.17)	153 (70.83)
18 months	69.4 ± 15.1	51 (23.61)	165 (76.39)
24 months	74.9 ± 15.8	46 (21.30)	170 (78.70)
p-value	<0.001	—	—
*Discharge values are estimated by interpolation between baseline and 6-month data.			

Surgical intervention was carried out in 68.52% (n = 148) of the patients, mostly via anterior approach (14.35%, n = 31), posterior spinal fixation (42.59%, n = 92), or a combination technique (11.57%, n = 25) (Table 3). Conservative measures, such as traction (7.41%, n = 16) or immobilization and bracing (24.07%, n = 52), were used in 31.48% (n = 68) of patients.

**Table 3:** Acute management details

Management type	Category	n (%)
Surgical intervention	Total	148 (68.52)
	Posterior spinal fixation	92 (42.59)
	Anterior approach	31 (14.35)
	Combined anterior & posterior	25 (11.57)
Conservative management	Total	68 (31.48)
	Immobilization & bracing	52 (24.07)
	Traction	16 (7.41)

**Figure 1:**
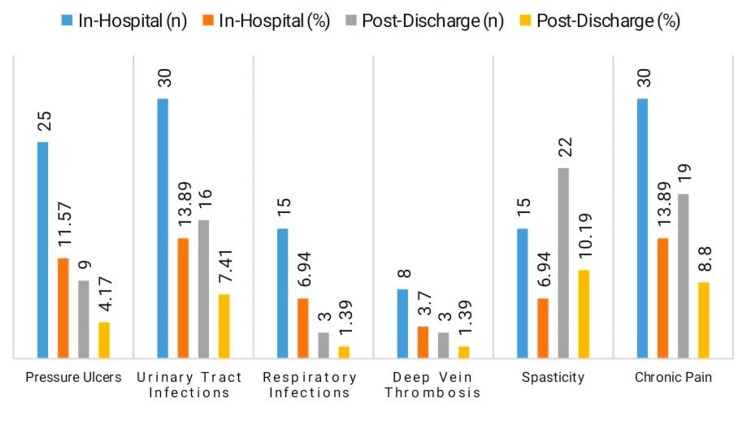
Complications by timing

Post-discharge and in-hospital complications were prevalent. A total of 11.57% (n = 25) of patients had pressure ulcers while they were in the hospital, and 4.17% (n = 9) after they were released (Figure 1). A total of 13.89% (n = 30) of patients had urinary tract infections while they were in the hospital, and 7.41% (n = 16) after they were discharged. Infections of the respiratory system were observed in 6.94% (n = 15) and 1.39% (n = 3). Chronic pain afflicted 13.89% (n = 30) and 8.80% (n = 19) of patients, whereas spasticity rose from 6.94% (n = 15) in-hospital to 10.19% (n = 22) post-discharge.

When it came to mobility, 18.52% (n = 40) continued to rely on wheelchairs, 42.59% (n = 92) ambulated with assistive devices, and 38.89% (n = 84) attained independent ambulation (Table 4). In terms of employment, 41.20% (n = 89) were jobless because of a handicap, 22.22% (n = 48) worked in modified work, and 36.57% (n = 79) went back to their prior jobs. The average length of hospital stay was 14.80 ± 6.70 days, with 20.83% (n = 45) remaining more than 21 days and 15.74% (n = 34) being released within seven days.

**Table 4:** Rehabilitation progress, social reintegration, and length of hospital stay (n = 216)

Variable	Category	Number of patients (n; %)
Ambulation	Independent ambulation	84 (38.89)
	Ambulation with device	92 (42.59)
	Wheelchair dependent	40 (18.52)
Occupational status	Returned to previous work	79 (36.57)
	Modified work	48 (22.22)
	Unemployed due to disability	89 (41.20)
Length of hospital stay (days)	≤7 days	34 (15.74)
	8–14 days	76 (35.19)
	15–21 days	61 (28.24)
	>21 days	45 (20.83)
	Mean ± SD (days)	14.8 ± 6.7

Significant associations were observed between level of injury and ambulation status (χ² = 12.34, df = 4, p = 0.015), surgical intervention and pressure ulcers (χ² = 9.78, df = 1, p = 0.002), comorbidities and urinary tract infections (χ² = 4.67, df = 1, p = 0.031), and occupational status and ambulation status (χ² = 14.52, df = 4, p = 0.006) (Table 5). Gender and mechanism of injury showed no significant association (p = 0.157).

**Table 5:** Associations between categorical variables using Chi-square test (n = 216) *p < 0.05 considered statistically significant

Variable 1	Variable 2	Chi-square (χ²)	Degrees of freedom (df)	p-value	Interpretation
Gender	Mechanism of Injury	5.21	3	0.157	Not statistically significant
Level of injury	Ambulation status	12.34	4	0.015*	Significant association
Surgical intervention	Pressure ulcers	9.78	1	0.002*	Significant association
Comorbidities (yes/no)	Urinary tract infection	4.67	1	0.031*	Significant association
Occupational status	Ambulation status	14.52	4	0.006*	Significant association

SCIM scores improved significantly at each follow-up interval (Table 6). For example, the mean difference between baseline and six months was 13.50 (SE = 1.20, p < 0.001), and between baseline and 24 months was 36.30 (SE = 1.60, p < 0.001). All pairwise comparisons indicated significant functional improvement over time, confirming progressive recovery in physical independence.

**Table 6:** Post-hoc pairwise comparisons of SCIM scores over follow-up periods with Bonferroni correction *p < 0.05 considered statistically significant

Time Point Comparison	Mean Difference (MD)	Standard Error (SE)	p-value (Bonferroni corrected)	Significance
Baseline vs. 6 months	13.5	1.2	<0.001*	Significant
Baseline vs. 12 months	23.2	1.4	<0.001*	Significant
Baseline vs. 18 months	30.8	1.5	<0.001*	Significant
Baseline vs. 24 months	36.3	1.6	<0.001*	Significant
6 months vs. 12 months	9.7	1.1	0.002*	Significant
6 months vs. 18 months	17.3	1.3	<0.001*	Significant
6 months vs. 24 months	22.8	1.4	<0.001*	Significant
12 months vs. 18 months	7.6	1.2	0.011*	Significant
12 months vs. 24 months	13.1	1.3	0.003*	Significant
18 months vs. 24 months	5.5	1.0	0.045*	Significant

## Discussion

Over the course of 24 months, the functional outcomes of 216 trauma patients with spinal injuries were assessed in this research, which showed notable neurological and functional recovery. By 24 months, the percentage of patients with less severe impairment (ASIA C/D/E) rose from 57.41% to 78.70%, whereas the percentage of patients with severe impairment (ASIA A/B) dropped from 42.59% at baseline to 21.30%. Motor and sensory subscores of ASIA were analyzed to provide a more detailed assessment of neurological recovery. This improvement is consistent with earlier research that showed neurological improvement in patients within two years after the injury, highlighting the possibility of significant recovery even in environments with limited resources [17].

Significant improvements in mobility, self-care, and sphincter control were shown by the rise in functional independence, as determined by the SCIM score, which went from a mean of 38.60 at admission to 74.90 after two years. These findings are in line with recent matched case-control research that demonstrated the importance of comprehensive rehabilitation programs for patients with spinal cord injuries [18]. Rehabilitation interventions, including physical therapy, occupational therapy, ambulation training, and aquatic therapy, were recorded and considered in the analysis, although protocols were not standardized. Furthermore, the slow but consistent increase in SCIM scores at six-month intervals (e.g., 52.10 at six months and 69.40 at 18 months) highlights the protracted recovery path that is common for individuals with spinal cord injuries. Recovery trajectories also varied by injury level (cervical, thoracic, lumbar), indicating heterogeneity in outcomes.

According to our research, 68.52% of patients had surgical intervention, mostly posterior spinal fixation. This incidence is consistent with other research showing a rise in surgical therapy in instances of traumatic SCI, mostly decompression procedures, which are a reflection of improvements in surgical technology and rising adherence to modern stabilizing methods [19]. Significantly, surgical intervention was linked to the development of pressure ulcers (p = 0.002), which is consistent with the prior study's findings regarding the recognized risk of immobilization and postoperative complications [20].

According to rehabilitation results, 38.89% of patients were able to walk independently again, while 42.59% of them required assistance equipment to walk, which is consistent with data from other comparable cohorts [21]. Occupational reintegration was difficult despite functional advances; only 36.57% of people returned to their prior jobs, and 41.20% of people were jobless because of their handicap. These outcomes highlight the impact of limited rehabilitation resources and socioeconomic barriers in low- and middle-income countries [22].

Urinary tract infection rates (13.89% in-hospital, 7.41% post-discharge) and chronic pain rates (13.89% and 8.80%, respectively) were similar to those reported in earlier research, which highlighted the importance of attentive post-acute care in preventing secondary complications that can impede the course of rehabilitation [23]. The study offers insightful information about the long-term functional outcomes of patients with spinal injuries in a Pakistani tertiary care setting. It emphasizes heterogeneity in recovery patterns due to varying injury levels and rehabilitation interventions, highlights context-specific challenges like high complication rates and limited occupational reintegration, and provides data consistent with global trends.

Strengths and limitations of the study

One of the study's advantages is its prospective design, which included a large sample size of 216 patients who were closely monitored over a two-year period. This allowed for a thorough assessment of neurological and functional recovery using standardized instruments, including the SCIM scores and the ASIA Impairment Scale with motor and sensory subscores. Multiple follow-up intervals and documentation of rehabilitation interventions allowed for a more detailed understanding of functional trajectories.

A comprehensive knowledge of patient outcomes in an actual tertiary care context is made possible by the inclusion of various follow-up intervals and evaluation of rehabilitation progress, complications, and social reintegration. The use of convenience sampling from a single location is one of the drawbacks; however, it might have an impact on how broadly the results can be applied. Furthermore, the interpretation of treatment results may be constrained by heterogeneity in injury levels, the observational nature of rehabilitation therapies, and the absence of standardized protocols and formal sample size calculation. Bias or variability in outcome measurement may be introduced by certain patients' dependence on telephone evaluations and loss of follow-up.

## Conclusions

Over a 24-month period, the research shows notable neurological and functional improvements in trauma patients with spinal injuries, including improvements in motor and sensory function, physical performance, and independence in daily activities. However, recovery trajectories varied by injury level and rehabilitation interventions, and challenges such as lingering complications and limited occupational reintegration persisted despite overall improvement. In order to maximize long-term results for patients with spinal injuries, especially in low-resource or low- and middle-income settings, our findings highlight the urgent need for standardized, context-specific rehabilitation programs, early intervention, and targeted management of comorbidities.
